# Recurrent Left Renal Fossa Clear Cell Renal Cell Carcinoma With Pancreatic Tail Invasion Presenting as Intestinal Obstruction

**DOI:** 10.7759/cureus.47953

**Published:** 2023-10-30

**Authors:** Mustafa Wasifuddin, Danelly J Gomez D' Aza, Akriti Pokhrel, Kapilkumar Manvar, Jen Chin Wang

**Affiliations:** 1 Internal Medicine, Brookdale University Hospital Medical Center, Brooklyn, USA; 2 Hematology and Medical Oncology, Brookdale University Hospital Medical Center, Brooklyn, USA; 3 Hematology and Oncology, Brookdale University Hospital Medical Center, Brooklyn, USA

**Keywords:** unilateral nephrectomy, post-nephrectomy renal cell carcinoma, renal cell carcinoma (rcc), mechanical intestinal obstruction, clear renal cell carcinoma

## Abstract

Distant renal cell carcinoma (RCC) metastatic disease is mostly seen in the lungs, bones, and lymph nodes. The incidence of local recurrences within the ipsilateral retroperitoneum (RFR) is very low. We report a case of a 79-year-old male with recurrent left renal fossa RCC with pancreatic tail invasion who presented with large bowel obstruction. To the best of our knowledge, no cases have been reported of recurrent left renal fossa RCC initially presenting as extrinsic large bowel obstruction.

## Introduction

Approximately 85% of all primary renal neoplasms are secondary to renal cell carcinomas (RCCs) originating in the renal cortex [1]. Tumors originating in the renal pelvis, namely, transitional cell carcinomas, comprise a further 8% [1]. Other infrequently occurring tumors include oncocytomas, parenchymal epithelial tumors, angiomyolipomas, renal sarcomas, and collecting duct tumors [1]. Distant clear cell renal cell carcinoma (ccRCC) metastatic disease is mostly seen in the lungs, bones, and lymph nodes. The incidence of local recurrences within the ipsilateral retroperitoneum (RFR) ranges between 0.8% and 3.6% [2]. Typical presentations include hematochezia and melena, weight loss, or no symptoms at all [3]. The notion that RCC is extremely resistant to classical fractionated radiation therapy (RT) is changing and the role of RT is now being considered as part of the treatment strategies in ccRCC. We report a case of a 79-year-old male with recurrent left renal fossa RCC with pancreatic tail invasion. He presented with an extrinsic large bowel obstruction that required surgical intervention. He was discharged to follow up to assess the role of RT after three months of systemic treatment. In the literature, only 10 cases of metastatic ccRCC to the intestine causing bowel obstruction have been reported [4-6]. These occur mostly secondary to intussusception, but no previous cases of recurrent left renal fossa RCC initially presenting as extrinsic large bowel obstruction have been reported. From our extensive review of the literature, this is the first case of recurrence presenting as an extrinsic intestinal obstruction. After appropriate surgical intervention, the patient was offered immunotherapy treatment followed by RT. Treatment strategies for ccRCC are evolving, and RT should be considered a part of the treatment regimen.

## Case presentation

A 79-year-old male with a past medical history of end-stage renal disease on hemodialysis, hypertension, atrial fibrillation, PT3C-Gleason 4+5 adenocarcinoma of the prostate (treated with radical prostatectomy and adjuvant androgen deprivation therapy) with biochemical recurrence, and left RCC T3AN0M0 (treated with left radical nephrectomy) presented to the ER with complaints of abdominal pain for two days. The pain was associated with constipation, nausea, bilious vomiting, loss of appetite, and small, hard stools with reduced caliber. The patient was afebrile and hemodynamically stable and looked uncomfortable. The abdominal exam showed a healed midline scar, left lateral flank incision, and left lower quadrant transverse incision. The abdomen was distended but non-tender. The rest of the exam was negative. Laboratory evaluation showed a white blood cell (WBC) count of 11.9 x 103/uL, hemoglobin of 10 g/dL, blood urea nitrogen of 38 mg/dl, creatinine of 9.6 mg/dl, and potassium of 4.2 mmol/l (Table 1).

**Table 1:** Laboratory workup of the patient.

Parameter	Patient values	Normal values
WBC	11.9 x 10^3^	4.5-11 x 10^3^
Hemoglobin	10 g/dL	Male: 13.8-17.2 g/dL
Blood urea nitrogen	38 mg/dL	6-24 mg/dL
Creatinine	9.6 mg/dL	Male: 0.7-1.3 mg/dL
Potassium	4.2 mmol/L	3.5-5.2 mmol/L
Prostate-specific antigen	9.5 ng/ml	<4.0 ng/mL
Lactate dehydrogenase	364 u/L	140-1280 u/L

**Figure 1:**
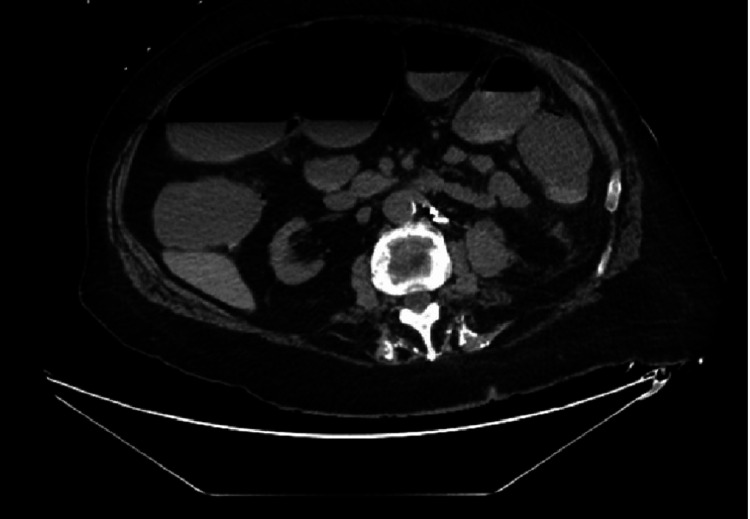
CT angiogram revealed 8 cm constricting lobular soft tissue with high-grade obstruction of the proximal large bowel.

**Figure 2:**
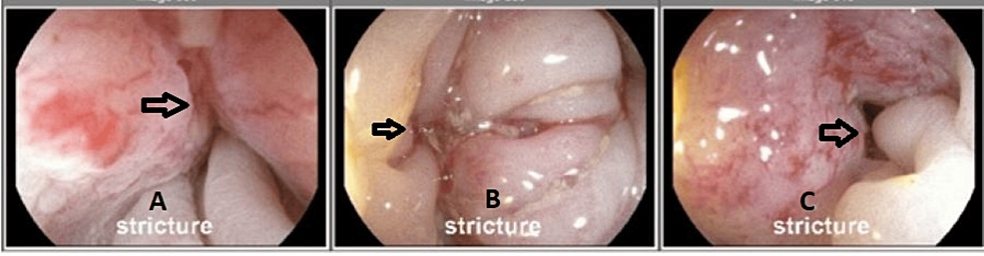
Sigmoidoscopy showed complete obstruction at 45 cm concerning ischemia/extrinsic compression, and no luminal mass was noted. Figures A, B, and C are shown from left to right with arrows pointing to the obstruction.

**Figure 3:**
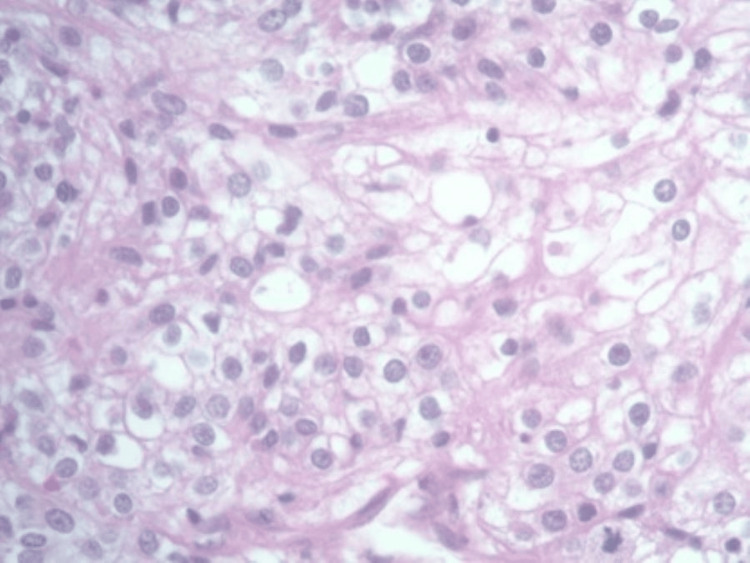
Original clear cell renal cell carcinoma showing sheets of clear cells with mild pleomorphic nuclei, prominent nucleoli, and abundant clear cytoplasm.

**Figure 4:**
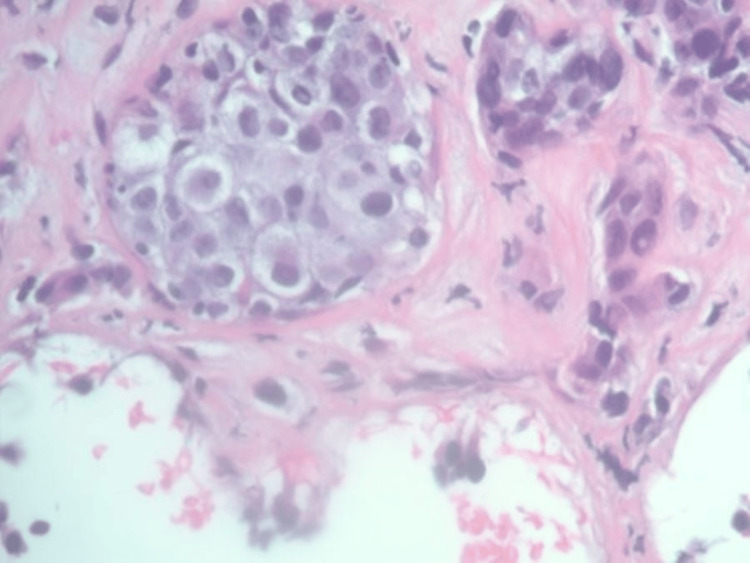
Metastatic clear cell renal cell carcinoma at left retroperitoneal site with nests of clear cells with hyperchromatic nuclei, moderate pleomorphic nuclei, and abundant basophilic cytoplasm infiltrated within fibrous tissue.

**Figure 5:**
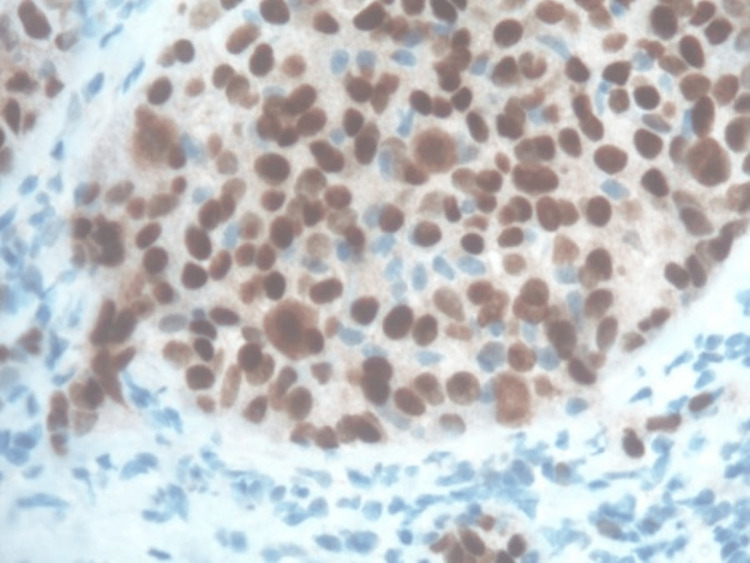
Metastatic clear cell renal cell carcinoma. The neoplastic cells are positive for PAX8, carbonic anhydrase 9, and vimentin and negative for CK7, NKX3.1, PSMA, and RCC. PSMA: prostate-specific membrane antigen; RCC: renal cell carcinoma.

**Figure 6:**
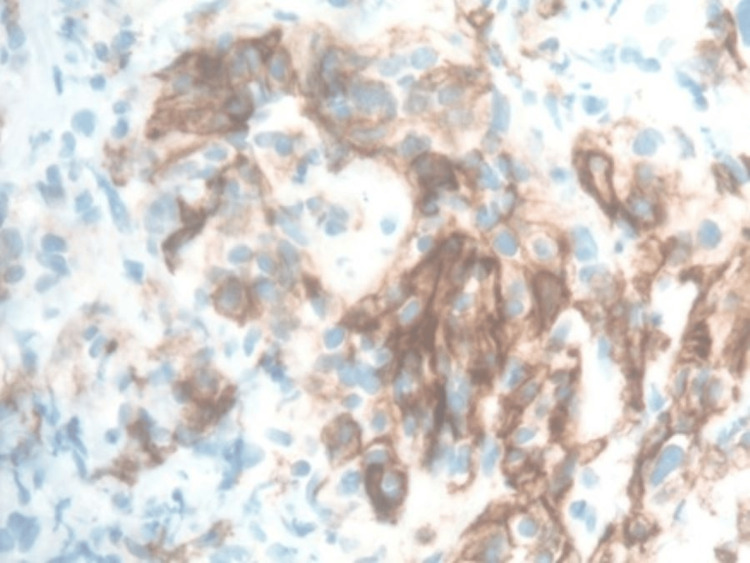
Neoplastic cells are negative for CK7, NKX3.1, PSMA, and RCC. PSMA: prostate-specific membrane antigen; RCC: renal cell carcinoma.

Prostate-specific antigen (PSA) was elevated to 9.5 ng/ml, consistent with previously elevated PSA. Lactate dehydrogenase was within normal limits at 364 U/l. CT angiogram revealed an 8 cm constricting lobular soft tissue mass in the proximal descending colon resulting in a high-grade obstruction of the proximal large bowel. The mass invaded the tail of the pancreas (Figure 1). The patient was initially managed conservatively with intravenous fluid resuscitation. A sigmoidoscopy of descending colon was performed, which showed evidence of prior colonic surgery at 25 cm from the anus and complete obstruction at 45 cm, with segmental mucosal edema and friability concerning extrinsic compression (Figure 2). No luminal mass was noted. The patient underwent a diagnostic laparoscopy. No surface liver metastasis was seen, and no peritoneal carcinomatosis was seen. Extensive laparoscopic adhesiolysis, small bowel resection with primary anastomosis and partial omentectomy, and the creation of loop transverse colostomy were performed. CT-guided tissue biopsy of the retroperitoneal mass was obtained. Biopsy showed invasive carcinoma consistent with renal origin (Figures 3, 4). Immunostaining for further characterization revealed the tumor cells to be positive for PAX-8, negative for renal cell antigen, negative for NKX3.1, positive for CAIX, positive for vimentin, and negative for CK7 (Figures 5, 6). The clinical history, histomorphology, and immunostaining supported the diagnosis of ccRCC. CT scan of the chest, abdomen, and pelvis with contrast was obtained for staging (T3aN0M0) and revealed no masses or nodules in the lungs. It showed post-surgical left nephrectomy with soft tissue mass in the left renal fossa contiguous with the pancreatic tail.

The case was presented on the tumor board. Imaging and pathology were reviewed in detail. Systemic chemotherapy was planned to be started outpatient after chemo port placement for local recurrence of ccRCC. Radiation oncology will follow the patient to see the role of radiation therapy after at least three months of systemic treatment and the patient will be started on androgen deprivation therapy (ADT) as an outpatient.

## Discussion

RCC is the most common kidney malignancy, accounting for 85% to 90% of these tumors. More than 50% of patients who undergo primary tumor resection have a remote recurrence. RCC can metastasize to any site in the body; however, metastases to the gastrointestinal (GI) tract are extremely rare, with an incidence as low as 0.7% [4]. A lack of clinical attention to GI metastases is given as they are presumed to be a feature of generalized metastatic disease. This has led to their underdiagnosis. Most RCCs are hypervascular, with clear cells being the most common histological subtype. They most commonly cause GI bleeding when they metastasize to the GI tract, according to a study conducted by Park et al., occurring in nearly 67% of patients. Common clinical symptoms include melena and hematochezia (66.7%), abdominal pain (13.3%), and weight loss (6.7%). Recurrent ccRCC rarely presents as bowel obstruction (6.7% of patients) [3]. Bowel obstruction primarily occurs in the form of intussusception, secondary to local spread or recurrence. A review of the literature yielded 10 cases of RCC causing intestinal obstruction [2,4-6]. In the majority of the reports, obstruction was most commonly secondary to intussusception. Our patient presented with abdominal pain, nausea, and bilious vomiting - all signs of intestinal obstruction. The initial presentation of ccRCC can be variable; therefore, bowel obstruction should be taken into account in RCC patients presenting with these symptoms. In malignant bowel obstruction (MBO), extrinsic, intramural, and/or intrinsic patterns are seen. Extrinsic compression of the bowel lumen occurs as a result of growing tumor burden, masses, or postirradiation fibrosis. Intramural compression results from the invasion of the bowel wall from a laterally growing tumor causing poor motility and from primary and metastatic lesions that occlude the lumen or act as lead points for intussusception. Vomiting has been observed to occur earlier in proximal bowel obstruction, whereas it occurs later in large bowel obstruction. Proximal bowel obstruction also results in intense, crampy pain patterns occurring at shorter intervals, whereas distal bowel obstruction is associated with less intense, deeper pain occurring at longer intervals. The diagnosis of MBO is based on historical and clinical findings; however, imaging is usually used for confirmation. CT studies with bowel contrast are implemented to identify the site of obstruction and evaluate dysmotility or partial obstruction. The slow passage of contrast through a nondilated bowel with no identifiable point of obstruction may signify a dysmotility disorder [7]. Our patient presented with abdominal pain, constipation, nausea, and vomiting. CT scan with contrast findings of the bowel showed a constricting soft tissue lobular mass. Endoscopic findings revealed no luminal mass, with complete obstruction at 45 cm, segmental mucosal edema, and friability concerning extrinsic compression.

Local recurrence following nephrectomy is a rare event. A study from the Mayo Clinic reported a local recurrence rate of only 1.8% in 1737 patients who had undergone nephrectomy with localized RCC [8]. Local recurrences involve the retroperitoneal structures such as the renal fossa and adrenal or axial musculoskeletal structures. Another study by Speed et al. observed the recurrence rates to be between 3% and 9% [9]. Our patient developed local recurrence in the renal fossa. Metastases to the pancreas are a very rare occurrence in RCC, with a reported incidence as low as 1.6%. The primary mechanism of spread for these metastases is through the hematogenous route [10]. There have been no reports of local invasion of the pancreas by RCC, to the best of our knowledge. Our patient presented with a direct invasion of the pancreatic tail. An explanation for this could be the seeding of malignant RCC tissue during the initial nephrectomy.

It is important to identify, standardize, and employ surveillance strategies following the treatment of localized RCC. A better characterization of clinical, histopathological, and CT findings can aid in the prediction of tumor recurrence and therefore time management. Incorporation of these factors in the stage-based stratification may play an important role in the identification of ccRCC tumor recurrences and treatment strategies [3,11]. T1 (tumors found only in the kidney and <7cm in size) tumors generally recur between 38 and 45 months, whereas T3 tumors generally recur between 17 and 28 months following initial nephrectomy - highlighting the important role played by the tumor stage in the timing of recurrence. Depending on the site of metastasis, different parameters are assessed. For local recurrence, identification of symptoms, careful history, physical exam, and abdominal CT scans are critical because resection of the renal fossa is associated with improved overall survival, which is the percentage of people alive at a certain point in time after a cancer diagnosis. The same applies to distant metastatic disease, except radiological imaging is directed toward the site of involvement [12].

Treatment strategies for RCC are evolving and the combination of immunotherapy and radiation therapy may be considered a part of treatment regimens. This combined strategy may result in a phenomenon known as the abscopal effect, which is a systemic antitumor effect achieved distant to a radiation treatment site [13]. The abscopal effect occurs when radiation treatment shrinks the target tumor and shrinkage of other untreated tumors in the body [13]. Surgical resection is still the mainstay of treatment for this disease. However, the risk of recurrence is high (between 20% and 40%) in patients with high-risk locoregional RCC who undergo nephrectomy only [12]. Adjuvant therapy with pembrolizumab, a programmed cell death protein 1 (PD-1) immune checkpoint inhibitor (ICI), was shown to improve disease-free survival vs. placebo in patients with RCC classified as intermediate-high or high risk of recurrence following nephrectomy as part of the KEYNOTE-564 trial [14]. In patients with recurrent RCC, salvage nephrectomy and thermal ablation have been proven to be effective [14]. In patients with metastatic ccRCC, the American Society of Clinical Oncology (ASCO) recommends ICI therapy in combination with vascular endothelial growth factor receptor (VEGFR) tyrosine kinase inhibitor (TKI) in patients with favorable-risk disease. A doublet regimen was recommended for patients with intermediate or poor risk and monotherapy with either an ICI or a VEGFR TKI in select patients [15]. According to the guidelines set by the National Comprehensive Cancer Network (NCCN), recurrent ccRCC is treated with cytoreductive nephrectomy when possible, followed by targeted therapy and/or immunotherapy. Preferred regimens for favorable-risk patients include axitinib (anti-vascular endothelial growth factor (anti-VEGF)) with pembrolizumab, cabozantinib (TKI) with nivolumab (IgG4 monoclonal antibody that blocks PD-1), or lenvatinib (anti-VEGF) with pembrolizumab.

## Conclusions

RCC has a myriad of clinical presentations. Intrinsic and extrinsic intestinal obstruction should be considered as an initial presentation of recurrent RCC, as seen in our case. Operating with a high degree of suspicion in patients with a history of RCC is important in identifying recurrence. Surveillance strategies following the treatment of localized RCC need to be identified and standardized and must be employed to identify recurrences. Treatment strategies for RCC are evolving and radiation therapy in combination with immunotherapy plays an important role in addition to surgery in the management of these patients.
